# Supplementary material for: Applying Flow Virometry to Study the HIV Envelope Glycoprotein and Differences Across HIV Model Systems
Source: Viruses. 2024 Jun 9;16(6):935. doi: 10.3390/v16060935 (PMC11209363; doi:10.3390/v16060935)
Supplement: Supplementary file 1 [file viruses-16-00935-s001.zip › Supplemental Figures (S1 - S6)_revised.pdf]

Figure S1. Anti-Env staining on cell culture supernatants from uninfected H9 T cells.

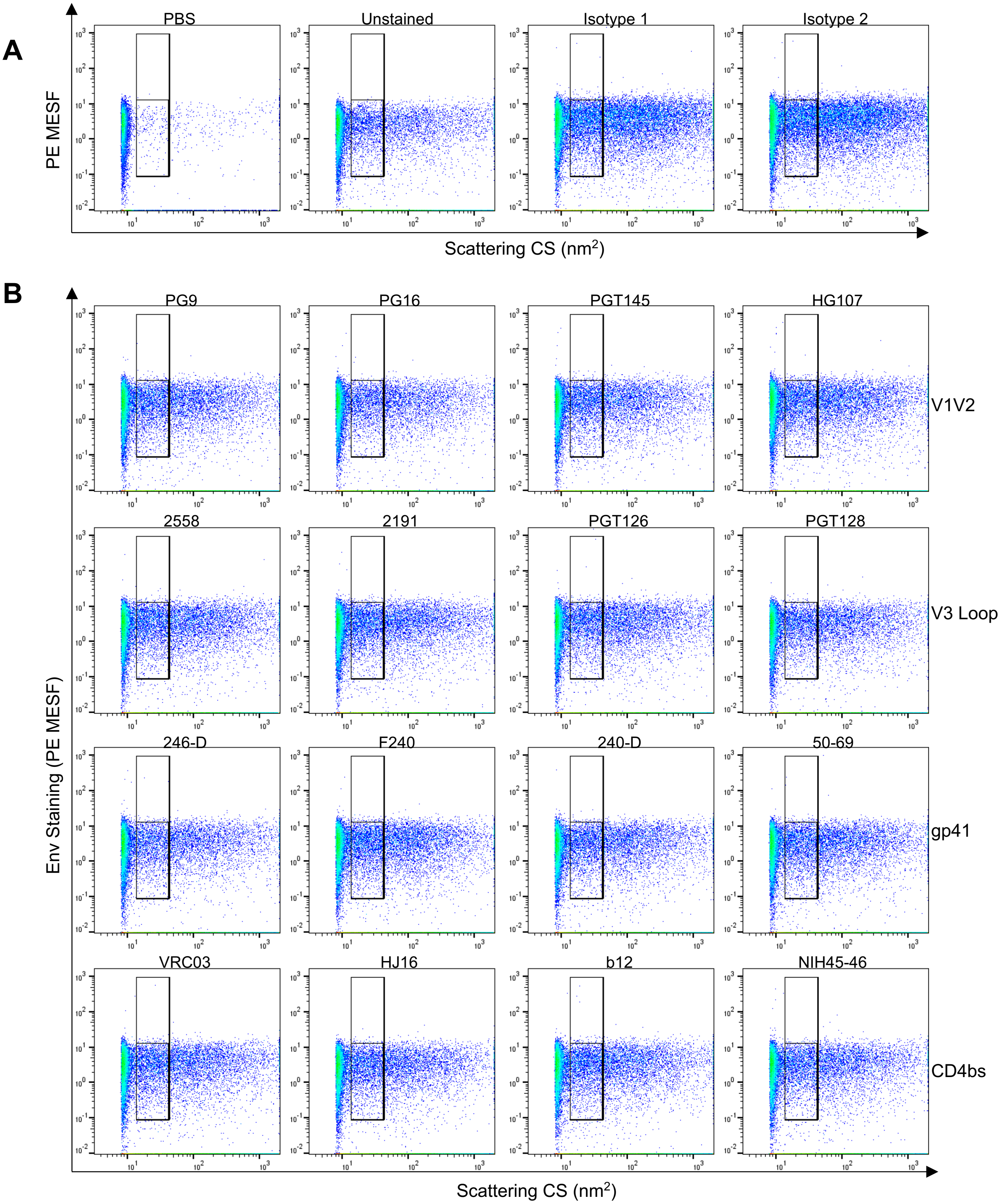

Figure S1. Anti-Env staining on cell culture supernatants from uninfected H9 T cells. (a) Dot plots displaying flow virometry controls assessing PBS alone, unstained culture supernatant, and supernatant stained with two different anti-human isotype control antibodies. Gating strategy is described in Figure 1. Positive staining is shown in the upper gate, while background levels fall within the lower gate as determined using the isotype controls. (b) A selection of antibody staining of antibodies targeting the variable loops 1 and 2 (V1V2), V3 loop, gp41 or the CD4bs.

Figure S2. Anti-Env staining on uninfected cell culture supernatants.

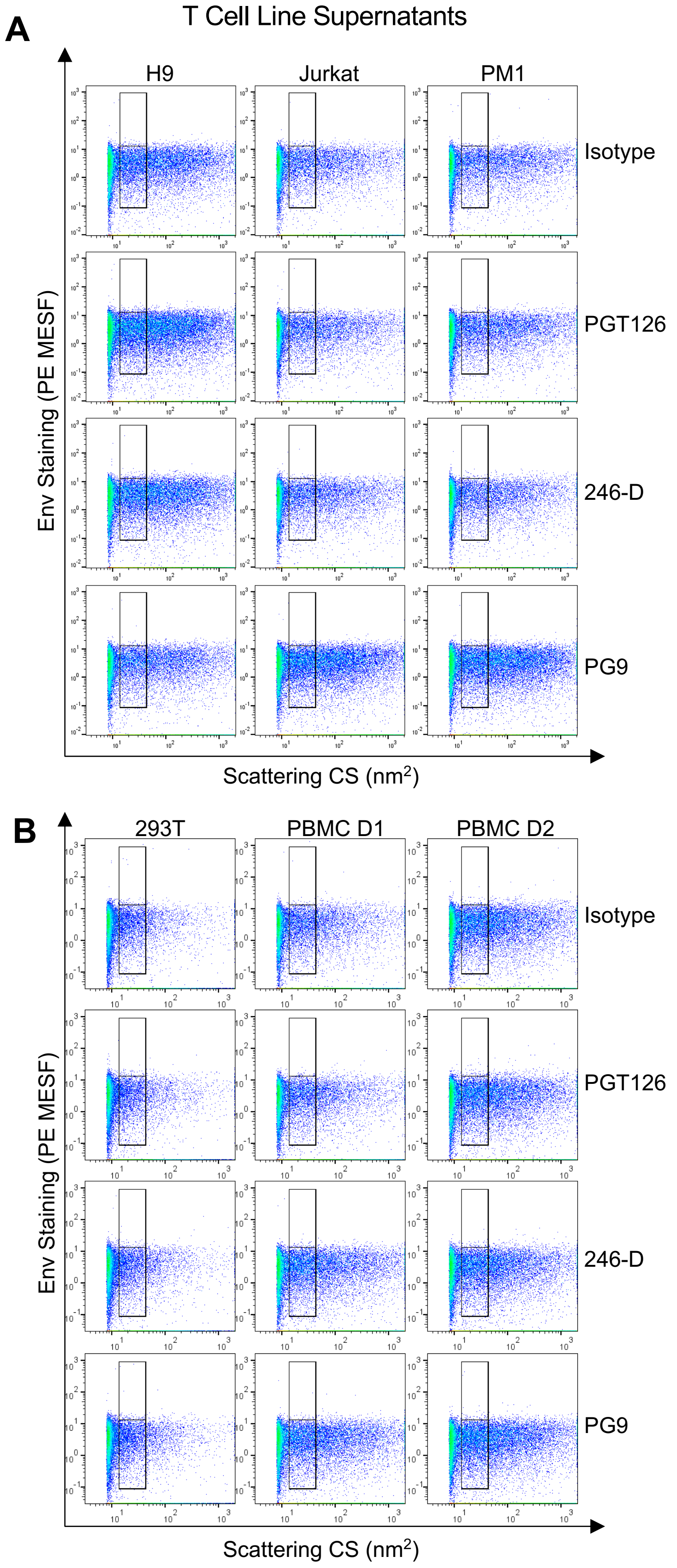

Figure S2. Anti-Env staining on uninfected cell culture supernatants. (a) Dot plots displaying matched flow virometry controls assessing staining of uninfected cell culture supernatants from T cell lines used to produce virus in Figure 2 (H9, Jurkat, PM1). Positive staining is shown in the upper gate, while background levels of staining fall within the black lower gate as determined using the isotype controls. (b) Staining of uninfected cell culture supernatants from 293T cells or two different PBMC donors (D1, D2).

Figure S3. Characterization of HEK293T-derived pseudoviruses.

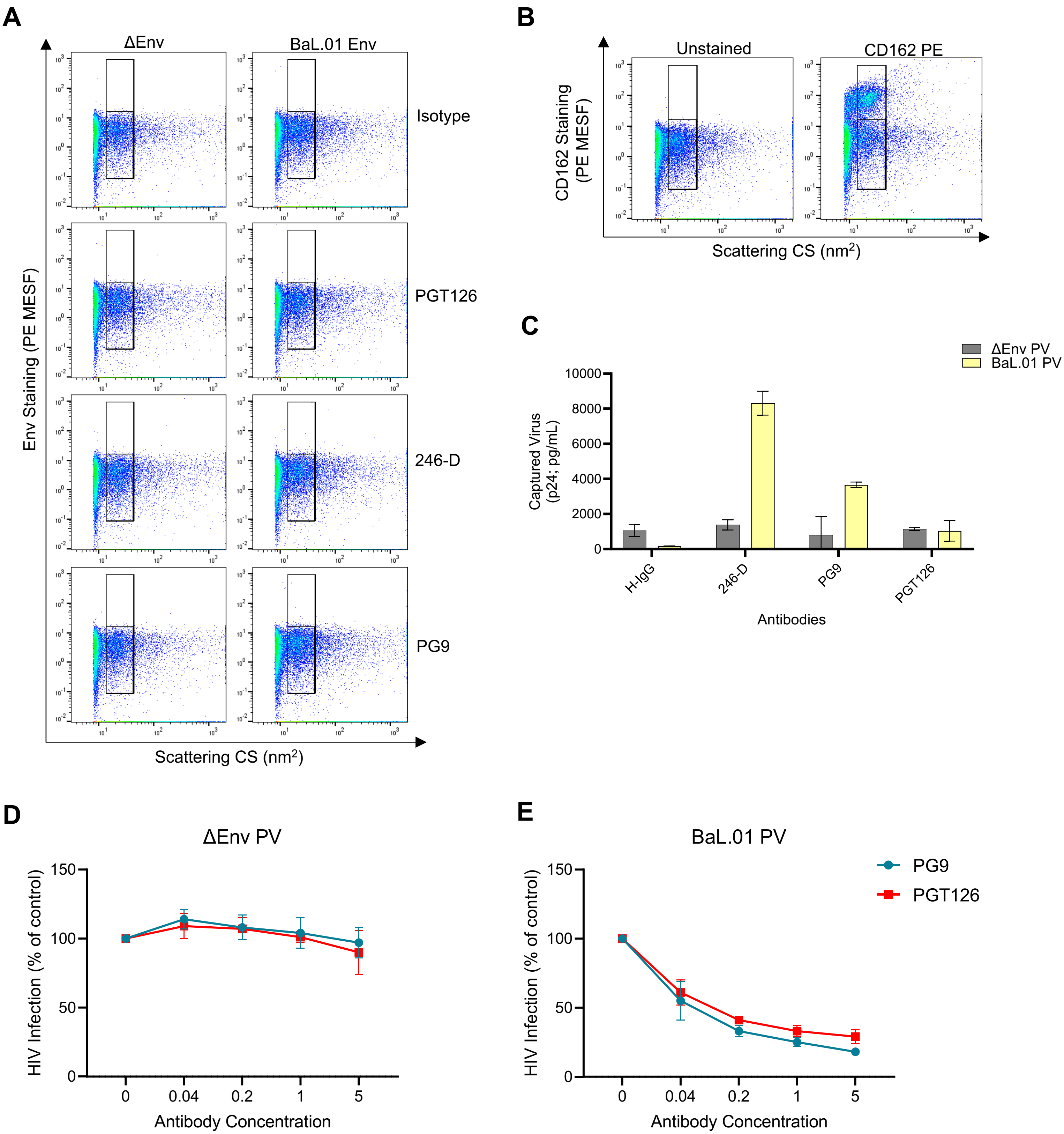

Figure S3. Characterization of HEK293T-derived pseudoviruses. (a) Dot plots displaying anti-Env antibody staining (PGT126, 246-D, PG9) of wild type pseudoviruses (no host proteins) produced in the HEK293T cell line through co-transfection of the BaL.01 envelope and SG3Δenv plasmids. (b) Validation of staining of CD162+ pseudovirus made in the same transfection as viruses from (a). (c) Plate-based capture of viruses using mAbs from (a). Viral capture was read out using p24 AlphaLISA. Data are representative of two independent experiments performed in duplicate. (d) Neutralization sensitivity of pseudoviruses without (ΔEnv PV) or with an HIV BaL.01 Env (e) as determined by neutralization assays with PGT126 and PG9. The luminescence reading when virus was added without antibody present (virus control) was set at 100%, with negligible raw luminescence values detected for the Δenv PV (d), as expected . Data are representative of one experiment tested with triplicate wells.

Figure S4. Bead-based anti-Env capture of T cell line-derived viruses.

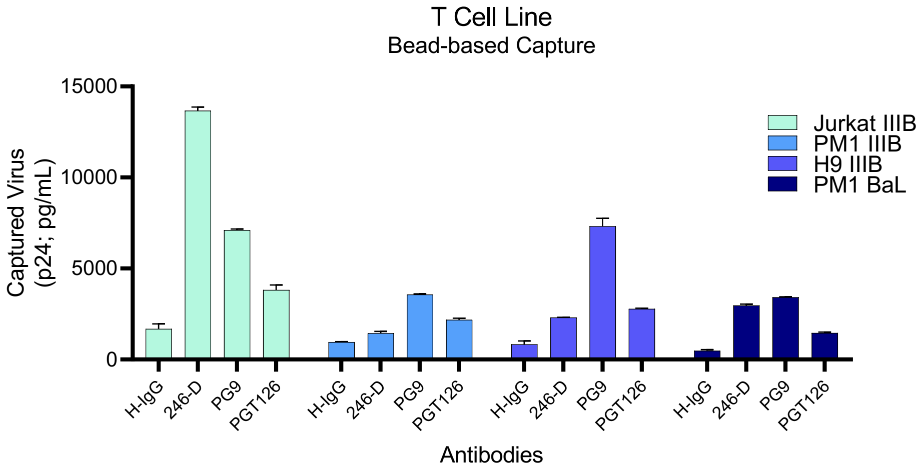

Figure S4. Bead-based anti-Env capture of T cell line-derived viruses. Normalized concentrations of viruses (IIIB and BaL; 50 ng/mL of p24) produced in T cell lines (PM1, Jurkat, H9) were incubated with immunomagnetic beads armed with the same antibodies from Figure 3. Viruses were lysed and HIV-1 p24 Gag was quantified using p24 AlphaLISA as an indicator of the amount of virus capture. Results show the mean ± SD of lysates tested in duplicate wells.

Figure S5. Comparing direct versus indirect staining on uninfected H9 cell culture supernatants.

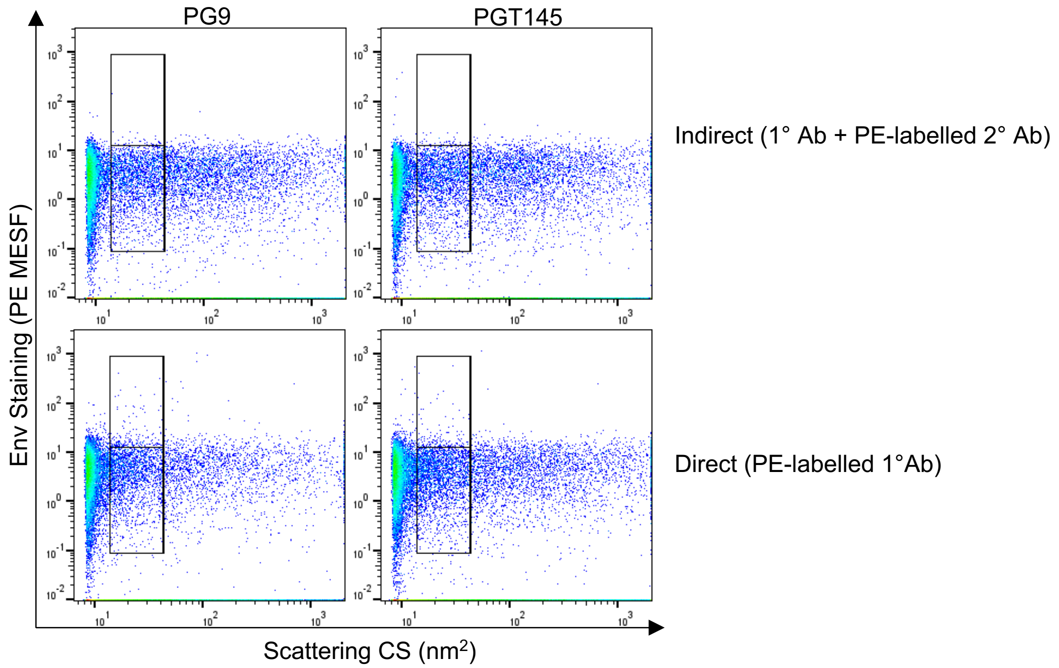

Figure S5. Comparing direct versus indirect staining on uninfected H9 cell culture supernatants. Indirect staining of uninfected H9 cell culture supernatants with unlabelled primary anti-gp120 antibodies (PGT145 and PG9) and a PE-conjugated secondary antibody (top panel). Direct staining of supernatants using PE-labelled PGT145 and PG9 antibodies (bottom panel). Positive staining is shown in the red upper gate, while background levels fall within the black lower gate.

Figure S6. Comparing different commercially available secondary antibodies for flow virometry staining.

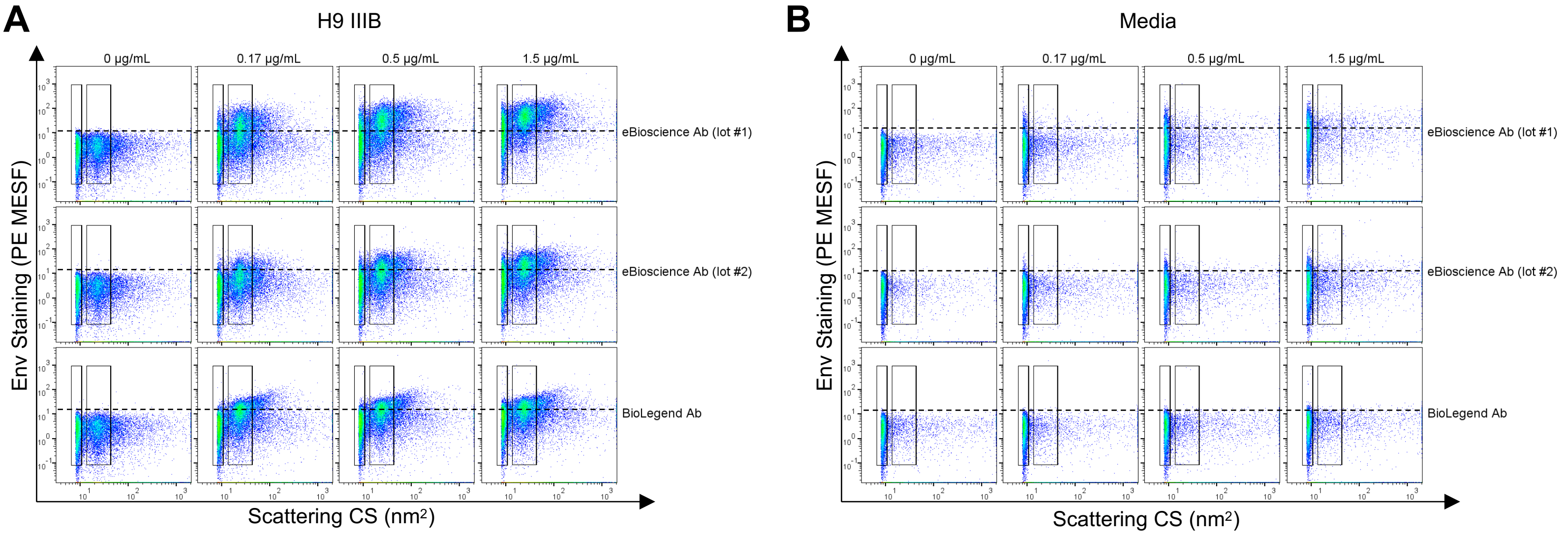

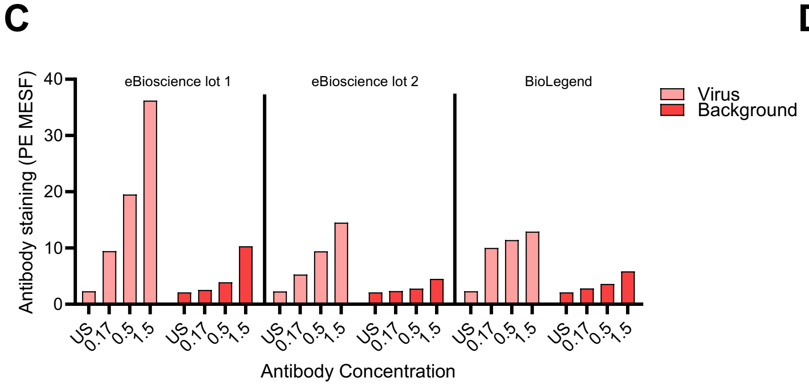

D

2° Ab	Ab Conc (µg/mL)	Virus : Background Staining Ratio
eBioscience (lot #1)	0.17	3.72
	0.5	**4.99**
	1.5	3.51
eBioscience (lot #2)	0.17	2.25
	0.5	**3.38**
	1.5	3.22
BioLegend	0.17	**3.57**
	0.5	3.14
	1.5	2.21

Figure S6. Comparing different commercially available secondary antibodies for flow virometry staining. (a) Dot plots displaying viruses stained with one anti-gp120 antibody (PG9) and various concentrations (0-1.5 µg/mL) of three different PE-labelled secondary antibodies (eBioScience lot #1 and 2, BioLegend). Dotted lines show levels of background fluorescence, determined using unstained samples. The leftmost staining gate denotes background at the cytometer threshold while the rightmost gate denotes the viral population. (b) Dot plots displaying staining of cell culture medium (RPMI) as in (a). (c) Graphical depiction of quantitative data from the gated regions in (a). (d) Table comparing the differences in the ratio of specific virus staining to background noise at the various concentrations of secondary antibody tested. Bolded text represents the optimal ratio for each antibody (i.e., highest specific staining and lowest viral noise).
